# Remarks on Irritative Fever, Commonly Called the Plymouth Dock-Yard Disease

**Published:** 1826-01

**Authors:** 


1. *Remarks on Irritative Fever, commonly called the Plymouth Dock-yard Disease; with Mr. Dryden's detailed Account of the Fatal Cases, including that of the lamented Surgeon, Dr. Bell.* By JOHN BUTTER, M.D. F.R.S. and W.S. &c. &c. 8vo. pp. 302, 1825.
2. *Cases of the Fatal Disease which occurred during the last Autumn, in His Majesty's Dock-yard at Devonport, with Observations.* By E. TRIPE, Esq. Surgeon. *Lond. Med. Journ. August* 1825.
3. *Official Report transmitted to the Honorable Commissioners of Transports, &c. &c. respecting the Death of Dr. Bell.* By MR. DRYDEN.

IN the summer and autumn of 1824, several men lost their lives from trifling accidents received while at work in His Majesty's dock-yard at Plymouth; and among others, Dr. Bell, the surgeon of the yard, fell a sacrifice. These unusual occurrences, attended by so great a mortality, produced a strong sensation in the public mind there, and various were the rumours circulated in consequence. Such exaggerated statements appeared in the newspapers that the author of the printed volume before us (Dr. Butter) who was then travelling in Wales, "was led to infer that the disease was either locked-jaw, a modified species of plague, or a malady altogether anomalous and new in its character." Accordingly, on his return to Plymouth, he instituted enquiries, not only of Mr. Dryden, the assistant-surgeon of the dock-yard, but also of the widows and friends of the deceased artificers, respecting the disease, and the results are now introduced to the public in a volume of more than 300 pages.

It appears that, besides the cases detailed by Dr. Butter—about 16 in number, there were upwards of 250 men laid up from their duty in the said dock-yard, by reason of various hurts received between the 24th June, and the 31st December, 1824—but only 15 had *the disease* in its malignant form—of whom *twelve* died. The number of accidental wounds was not greater than usual during that period, but the fatality from such apparently trifling causes was unprecedented. It is calculated that, in the course of each year, between three and four thousand men wound or hurt themselves, in Plymouth naval yard, in such a manner as to apply for surgical assistance, but of that number only about *four hundred*, on an average, are incapacitated for duty. Mr. Dryden has been assistant-surgeon of the yard for ten years, and has known only two instances of men dying from the fever supervening on local injuries. There are at present about 2000 mechanics employed in the dock-yard, and liable to these accidents. Their work is from six in the morning till six in the evening, during summer—and from day light till dark, in winter. They take their meals at 8, 12, and 7 o'clock, living temperately.

The nature of the disease, of which the men in question died, admits of no doubt. It was that kind of fever, generally designated "irritative," which supervenes on dissection wounds, and on punctured or other kinds of wounds in bad constitutions. But the etiology of it is far from being settled, as our readers well know—some maintaining that (in dissection wounds at least) a morbific agent is introduced—while others maintain that the whole morbid phenomena depend on constitutional susceptibility at the time of the injury. Our readers are aware that we have always advocated the agency of a morbific matter in dissection wounds, while we admit that constitutional irritability alone will give rise to the phenomena in many cases. We shall first lay before our readers some of the cases detailed in this volume, and then proceed to a farther consideration of the nature and treatment of the disease.

The first case which we shall introduce (the second on Dr. Butter's list) is remarkable for its duration, and the vicissitudes of hope and fear engendered in its course.

"*Case* 2. John Henwood, æt. 50, house-carpenter, working as a sawyer, and residing at Mill-brook, dark complexion, robust and healthy.

"On the 4th of August, 1824, received a slightly lacerated wound on his right hand by a saw. Cataplasms applied. Cathartic pills and cathartic mixture,* taken immediately.

"5th. Easy.

"6th. Reported unable to attend the surgery, and on being visited, was found labouring under considerable fever. Pulse about 120, strong and incompressible; severe head-ache and thirst. The whole hand much inflamed. Blooded to 34 ounces; cathartic pills given, and compound powder of jalap.—*(Ph: Ed:)*

"7th, 8th, and 9th. Fever abated, cataplasms continued, and fomentations ordered.

"10th. Continues better, but the whole arm is much swollen and erysipelatous; an obscure fluctuation was perceived on the back of the hand, from which, on being opened, pus could be pressed out as from a sponge. Calomel, opium, and antimonial powder, given with confection in boluses.

"11th. The tumefaction of the hand much reduced. Anodyne bolus continued, and fomentations of poppy-heads and chamomile flowers.

"12th. The wound on the hand discharging freely pus and membranous sloughs. The arm very painful; pulse rapid, but soft; tongue clean and moist. Cataplasms, fomentations, and boluses continued.

"13th. Made an opening on the fore arm, which discharged a quantity of pus and sloughy matter.

"14th. Medicines continued.

"15th. Made another opening on the back and inferior part of the humerus, which discharged about eight ounces of pus.

"16th. The pain and tumefaction of the arm much reduced; the discharge very free, with quantities of sloughy matter. Bark given, with diluted sulphuric acid.

"17th. Fever much abated; supported the arm with a bandage leaving the apertures clear, to which poultices were continued. Wine given; bark, &c. repeated.

---

"* The cathartic pills and mixture, directed in the foregoing formulæ, may be considered as those which were generally employed in the Dock-yard, when a difference is not otherwise expressed."

"18th. Better—matter could be pressed along the humerus from behind the scapula. The axillary glands not affected. Cathartic mixture taken occasionally.

"19th and 20th. Improving.

"26th. The hand and fore arm nearly sound, and the discharge from the humerus much lessened; complains of pain in his left leg and thigh, which are œdematous, but not discoloured.

"September 1st. The left thigh very painful; body much emaciated; fever increased. Bark omitted; anodyne bolus given at night, and cathartic mixture occasionally; fomentations and soap liniment ordered.

"3d. Symptoms as above; pulse about 130, and weak; tongue moist; no appetite. Fomentations continued.

"7th to 10th. Fluctuation of matter became gradually perceptible in different parts of the left thigh, to which cataplasms were applied. His strength was much exhausted.

"12th. The propriety of opening the limb doubted, from his excessive debility.

"15th. By Dr. Bell's consent, an incision was made a little above the patella, and another on the posterior part of the thigh, from which was discharged an immense quantity of well-formed pus; he bore the operation well. Wine and opium continued.

"16th. Discharge very profuse; the tension and pain of the limb much relieved.

"18th. Feels more comfortable, but evidently loses ground. Medicines, &c. repeated.

"20th. A slough formed on the lower part of the sacrum, and excoriations in several places over the spine. Cataplasms of yeast were applied.

"25th. A large slough separating from the back part of the thigh. Bark, wine, and opium, continued.

"October 2d. Debility so great that it is with much difficulty he can be moved to apply the dressing; gangrene commencing on the right heel. He lingered until the 11th, when he sank one of the most miserable objects ever beheld. Body not examined." 9.

In the above case it will be observed that, on the second day after the injury, the hand inflamed, and the constitution sympathised with the local injury, as evinced by head-ache and hurried circulation, with other febrile phenomena. He was bled to 34 ounces, and it appears that the symptoms were mitigated during the three succeeding days. On the 6th day after the injury, however, the whole arm was erysipelatous, and when an opening was made into the cellular substance matter could be squeezed out, as from a sponge. By this the tension was relieved, and an amendment was experienced. Bark and acid were given on the 12th day after the accident, and for a space of 14 days the amendment continued. Then a second train of unfavourable symptoms occurred, which went on from bad to worse, till at length he was worn out by complicated miseries, and died on the 68th day after the laceration of the hand.

Dr. Butter attributes the temporary mitigation of symptoms entirely to the incisions, and gives the venesection no credit. In this point we disagree with our author. We are no advocates, as our readers know, for excessive or indiscriminate depletion in diseases of this character; but in a robust and healthy man, with a pulse "*strong and incompressible*," we do maintain that venesection to 34 ounces was a measure, not only justifiable but highly proper, under the existing circumstances, and we are convinced that this timely depletion had a very principal share in circumscribing the extent and amount of the local inflammation.

The next case which we shall introduce was one of the very few which terminated successfully.

"*Case* 2. (Case 4 of Dr. B.) WILLIAM BUTTERS, æt. 40, a joiner, and a very healthy man, possessing an excellent constitution, who has been always most temperate in his living, residing in Chapel-street, Stonehouse, with his wife and six children.

"On Tuesday, the 10th of August, 1824, whilst planing a piece of mahogany in the joiner's shop of his Majesty's Dock-yard at Devonport, he tore up the nail of his right ring finger by a splinter of wood. Dr. BELL removed the nail on his application at the surgery, and ordered poultices, which were continued to the finger for five days, and afterwards light dressings for three days, he, BUTTERS, continuing at his work during that time.

"This accident happened the more easily in consequence of the infirm state of the nails of the ring and middle fingers of the right hand, owing to a fracture of the last phalanges, produced by their being jammed between two pieces of timber in February last. He was then confined six weeks by these fractures, but suffered no unusual constitutional irritation.

"On Wednesday morning, 18th of August, whilst dressing himself, he felt shivered and unwell, but nevertheless went to the Dock-yard. Whilst there, he became so ill with general disorder, rigors, and faintness, that he was obliged soon to quit the yard previous to the usual hour of departure. He applied to Dr. BELL, who remarked that his illness could not depend on the finger, which, on this day, looked healthy and granulating. Poultices were again applied to the finger, and cathartic medicines ordered.

"19th. His bowels had been freely opened. He was somewhat easier.

"20th. His fever had increased, with severe head-ache. His hand and arm were now much inflamed: the sore felt cold and smarting, rendering the connexion, which had been before doubtful, between the local injury and the constitutional disturbance, both manifest and certain. V.S. ad ℥xxxiv.

"Medicam: cathartic: cum ant: tart: subinde. fotus partibus affectis.

"21st. The blood, drawn yesterday by Mr. Dryden, buffed; fever not diminished; head-ache unsubdued. His head was now so disordered, that a sort of redness *(photopsia)* prevailed before his eyes, and a phantom, as though a piece of wood was encircled by fire—signs highly indicative of great cerebral excitement. Hand and arm very painful, much swollen, and erysipelatous. The redness appeared in patches and lines on the inner side of the arm in the course of the absorbent vessels, which were red even to the axilla. Great œdema over the whole arm. Admoveantur hirudines xvi. temporibus.

"Repr: mistura cathartica.

"Sumat nocte bol: sequent:

"℞ Pulv: Antimon: gr. vi.
Ext: opii gr. ij. confect: q: s: m.

"22nd. The leeches afforded relief to his head. Symptoms as yesterday. Continuentur cataplasmata et fotus.

"23rd and 24th. No particular alteration.

"25th. The whole hand and arm still erysipelatous, and greatly swollen in an increased degree, pitting like dough on pressure, his pulse rapid and weak; strength greatly exhausted; head-ache rather lessened, but still very bad; tongue moist and clean. On the back of his right hand an obscure fluctuation was now perceptible, which Mr. Dryden opened by an incision nearly two inches in length; the cellular tissue was injected with pus.

"Habeat ol: ricini ℥i. statim.

"26th. The tension of the whole arm was lessened, and the head-ache relieved, after the opening, the redness disappearing.

"27th. Improving. Ol: ricini ℥i. st.

"28th, 29th, and 30th. Better in every respect. Repr: oleum. habeat mistur: cathartic: pro re natâ.

"September 2nd. Soft dressings and light bandages were re-applied to the finger and arm, in lieu of the poultices, &c. which had been continued up to this period over the whole limb.

"8th. Convalescent. Owing, however, to excessive debility, and stiffness of his right hand and arm, Butters could not return to his duty in the Dock-yard until Monday, the 11th of October." 20.

We have introduced this case to shew that recovery may take place where bleeding has been employed, and where stimulation has been withheld. It appears perfectly evident that the whole drift of Dr. Butter's work is to shew the injurious consequences of blood-letting in the disease under consideration: and, as this case tells rather against him, he endeavours, like a good lawyer, to weaken the adversary's strong points. Having sent for this man who survived after bleeding, he questioned him on certain subjects, and then makes the following remarks, which we cannot help thinking as not of the most ingenuous kind, however carefully they have been constructed, with a view to candour.

"*Patients may be allowed to form some opinion of the treatment practised on them.* BUTTERS says that he did not find any relief from venesection; that the leeches diminished his head-ache considerably; but that the incision into his hand produced the most decided amendment. To what other circumstances can this recovery be attributed? *To the leeches, therefore, as accessary, and to the incision, as principal,* if to any measures at all, would I ascribe the salvation of his life. But a great deal must be allowed for his most excellent constitution. I mention the fact merely, without questioning the propriety of the venesection employed in any instance, that this is the *only man* who recovered *after bleeding* from the arm." 23.

We have put some parts of the above remarks in Italics, and they will speak for themselves. The latter part of the passage is nearly as much as to say "I have known several people swallow an ounce of arsenic, and I never knew but one recover from such a dose—still I by no means wish to insinuate that an ounce of arsenic is an improper dose."

Some of the other cases we shall now abridge.

*Case* 3. (Case 5) R. Horne, aged 32 years, robust and tall, received a severe contusion from the fall of a piece of English oak on the great toe of the left foot, 30th August. Cataplasms—cathartics. 31st. Remained from work, but walked to the surgery. Sept. 1. Leg painful—towards evening shivering and feverish symptoms. 2nd. Fever established. Twelve leeches to the temples—cathartics—cataplasms. 3rd. Fever continues—pain in the left thigh—foot and ankle erysipelatous—some confusion of intellect—tendency to delirium. Twelve leeches more to the head—fomentations. 4th. Increase of fever—pulse 120 and strong—erysipelas extending up the leg—venesectio ad ℥xx. Calomel purgative. 5th. Fever abated. 6th. No alteration since yesterday. *Evening*—fever increasing—erysipelas extending—delirium—venesectio ad ℥xvi. At ten o'clock at night the venesection was again repeated, the head-ache being most severe, with attempts to get out of bed. 7th. An obscure fluctuation over the left foot. When opened, a bloody serum issued. Head-ache and fever lessened—exhaustion great—pulse 120 and weak—cathartic—opiate. 8th. Tension of the foot relieved—but sphacelus is forming below the external malleolus. 9th. Stupor—pulse 130—incision into the sphacelated parts—10th and 11th, nearly the same. 12th. The tension and inflammation of the whole limb diminished—the original wound on the great toe nearly well—

hope of recovery entertained—debility excessive. 13th. Pulse scarcely perceptible. 14th. Died. Dissection only disclosed the cellular substance every where distended with a yellowish serum—pus formed around the sphacelated spots, which had not extended deeper than the fascia—subjacent muscles sound.

*Case* 4, (6.) G. Nichols, aged 45, tall, stout, athletic man, received on the 7th, 8th, and 9th September, sundry slight abrasions over his left shin bone, the weather being very hot and sultry at the time. 11th. Did not work, but came to the surgery. Monday 13th. He performed his usual work in the Dock-yard, although the leg had become very painful. Had some rigors this day, which he attributed to the cold lotion applied to the leg. At night, red lines were seen in the thigh, with tenderness in the inguinal glands—flushed and feverish—pain in the occiput. 14th. Visited by Dr. Bell, and the disease pronounced to be the reigning endemic of the Dock-yard. Cataplasms and fomentations—emetic-cathartic. 15th. Pain extended from the leg to the groin, hip, and back—the absorbents exhibited a streaky redness, of an erratic character—considerable œdema of the whole limb—fever increased—pulse 110, and full—*venesectio ad* ℥xxxiv. *Calomel and antimony night and morning.* "16th. Fever lessened—redness of the limb scarcely apparent—tenderness in the groin nearly gone." *Castor oil—the calomel and antimony, after the operation of the oil.* 17th. Not worse—bowels open—*cathartic mixture, with tartarized antimony, every* 4 *hours.* 18th. Has passed a restless and dreadful night—pain in the right hypochondrium—pulse 110, and small. 12 *leeches to the side—purgatives repeated.* 5, P.M. The symptoms increased—pain and tenderness all over the abdomen—*venesectio ad* ℥xx. Syncope and hiccup followed the bleeding. *Castor oil—enemata*—24 leeches ordered to the abdomen, an anodyne draught. 19th. Leeches had not been applied, on account of the great restlessness—worse this morning—pulse scarcely perceptible. Died at 10 o'clock.

"*Dissection.* The body was examined eight hours after death. Emaciation trifling. Parietes covered with fat. Intestines generally inflamed, particularly about the termination of the ileum, and the commencement of the cæcum, which were dark in colour. The mesentery and meso-colon were vascular with red patches, the whole being amassed in sero-purulent fluid. The right kidney was completely disorganized, and changed into a mass, like thick cream in colour and consistence. The left kidney sound. The urinary bladder adhered very firmly to the surrounding parts, and contained about six ounces of healthy urine." The other viscera, particularly the liver, were healthy in appearance. 37.

The examination, in this case, "shewed a result which had been common, in the foregoing examples, viz. an inflammation of certain parts of the ileum and cæcum, bordering on the darkness of mortification, with red patches on the mesentery, &c. and a quantity of sero-purulent fluid (probably also coagulable lymph) in the abdomen." This statement is to be borne in mind by the reader of Dr. Butter's work, the *tendency* of which is to proscribe the lancet in toto, or nearly so, from the methodus medendi in this disease.

We shall be able to give room to but one more case—that of the unfortunate surgeon who had the charge of the Dock-yard, and whose mind was dreadfully harassed by the fatality that prevailed. Having procured a copy of the official statement made by Mr. Dryden to the Naval Medical Board, respecting Dr. Bell's illness and death, we have compared it with that furnished by the same gentleman, and now published by Dr. Butter. The discrepancies we have printed in Italics in the case, and alluded to between the brackets.

"*Case* 5. (9.) Dr. JAMES BELL, æt. 58, late Surgeon of his Majesty's Dock-yard at Devonport, rather stout, sometimes dyspeptic, but generally healthy:

"On the afternoon of Sunday, the 19th of September, 1824, attended the examination of GREGORY NICHOLS's body, and scratched his right fore finger, over the extensor tendon on the last phalanx, when he was directing a pupil how to sew up the body, with a surgeon's needle, which had been previously used for that purpose. At this time the Doctor appeared in his usual state of health, and took no particular notice of the puncture or scratch so inflicted on his finger.* ['The part now barely perceptible, and not inflamed,' in the original.]

"On the following morning, September 20th, Dr. BELL attended the Surgery at the accustomed hour of nine o'clock, and complained of feeling unwell. When washing his hands he first felt a smarting in the scratch over his right fore finger; whereupon he became shivered, and impressed with an idea that he had caught the disease, then prevailing among the Mechanics of the Dock-yard, which disease had previously agitated his mind, and now made him very apprehensive for his safety. He remained, however, at the office about an hour and a half, and by that time the rigors and general indisposition had increased in such a

"* It may be here remarked that, in consequence of the unprecedented fatality which had lately occurred, and the untoward character which the wounds generally assumed at this time, he underwent much bodily fatigue, as well as anxiety of mind; and that in crossing the water in a boat on Saturday, to visit a patient at Torpoint, he got thoroughly wetted with rain."

degree as to produce considerable alarm. Pulse 96, and full. Bowels opened by an aperient, taken on the preceding evening.* The wound on the finger was not inflamed. He was recommended *to lose blood from the arm*, [not in the original] and to take an emetic, to both of which propositions he had a great aversion, and made insuperable objections. He went to bed, however, before twelve o'clock at noon, and endeavoured to make himself sick by irritating the fauces with his fingers, and by drinking warm water. In the course of the day, his fever had increased, and a reaction or flushing followed the rigors. Dr. Dickson, Physician to the Royal Naval Hospital, at Stonehouse, and Dr. Yonge, of Plymouth, were consulted. He now consented to lose about sixteen ounces of blood from the left arm, by a small orifice, and to take the subjoined Bolus:—

" ℞: Hydr: Submur: gr. v.
Pulv: Ant: gr. iv.
Confect: q: s: M:

which was to be followed by some of the Cathartic mixture in the morning.† [' Great timidity about every measure adopted.']

" 21st. Passed a restless night, with an increase of fever. Pulse 130, and full; stomach very irritable; *nausea great;* [not in original] tongue coated; bowels open; complains of pain and tenderness over his right pectoral muscle *and shoulder, on which he cannot bear the least pressure; but the wound on the right fore finger is not at all inflamed.* [Not in the original MS.]

Rep$^{r.}$ V: S: ad ℥xxx.

Ordered effervescing draughts, with saline medicines, every second hour.

" *Eight o'clock, P. M. No relief from the second bleeding. The blood was neither buffed nor cupped, the coagulum was rather firm.* [Not in the original case.] Speaks of a distressing pain about his sacrum and loins, with general lassitude and great weight, and evinces considerable anxiety. *There still exists much soreness over the right pectoral muscle, and there is a great prostration of his muscular energies; but withal there is no head-ache.* [Not in the original.] Pulse 130, and *small.* [' *Soft*' in MS.]

" ' Hyd$^{r.}$ Submur: H.S.'

Bolus, et Contr$^{r.}$ Haust: effervesc: cum Potassæ Nitratis gr. v, 3tiis horis.

Illinatur Linimt: Saponis in partes affectas.

" 22d. Symptoms all aggravated. Passed a sleepless and distressing night, owing to the excruciating pain in his back, but felt relief towards morning, from profuse perspirations. *Still feels pain in his right shoul-*

" * The Doctor frequently took Pil: Hydr: and Ext: Colocynth: or small doses of Magnes: Sulph: to relieve his dyspeptic complaints."

" † As the medicines given to Dr. Bell, though judiciously directed, made no effectual check to the march of his disease, it is thought necessary to give a particular account, only so far as seems to be important in detail."

*der, with inability to move the arm;* [not in the original case] complains of indistinct vision, and mistakes the distance when he attempts to take any thing. Nausea continues; pulse 130, and irregular.

"Appr: Empl: Lyttæ Epigastrio;
Medicament: Contr.

"23d. Slept a little during the night; nausea alleviated, but not gone; back-ache mitigated; pulse very irregular, and frequent; articulation difficult; thoughts confused; *no pain in any part but the pectoral muscle, when pressed upon; there is no redness nor visible sign of inflammation on the skin; the scratched part of the finger is neither inflamed nor swollen* [not in the original case.] He took camphorated Mixture, with the Nitrous Spirit of Æther, every two hours, and Wine frequently.

"Towards evening his breathing became laborious, and delirium appeared.

"Appr: Empl: Lyttæ suris et imo Colli.

"24th. Became perfectly insensible during the night; pulse wavering, occasionally indistinct, and imperceptible; breathing laborious; stupor and coma present; the sphincters relaxed, and the discharges passed involuntarily; profuse perspiration towards morning, when the breathing improved. Took four doses of the Sulphate of Quinine (℈ i.) without benefit. His deglutition was at last impeded, and a vacant stare occupied his countenance until *seven o'clock on this evening*, when he sunk without a struggle. Body not examined.

"P.S. The Sulphate of Quinine has since been ascertained by Dr. DICKSON not to have been genuine." 64.

We have set forth the discrepancies in the two statements of Dr. Bell's case by the same reporter, not with the view of attaching any blame of inaccuracy to Mr. Dryden, or mala fides to Dr. Butter, for we distinctly disavow any such suspicions; but merely to shew that the printed case reads considerably more unfavourable to the antiphlogistic treatment than the manuscript and official report, as may be readily seen from a glance at the passages in italics. These circumstances are heightened by the collocation of the case with that of the late Mr. Dease of Dublin, already recorded in this Journal, and where the parallel is evidently drawn closer in consequence of the alterations from the original and official statement. We consider it our bounden duty to draw the attention of the public to this point, because the parallel, the history of Dr. Bell's case, and the subsequent remarks of Dr. Butter, are all calculated to proscribe a measure which, although we believe to be often, perhaps generally, carried too far in the disease under consideration, cannot, at the same time, be banished from practice, without great detriment to the patient. We have repeatedly urged in this Journal the caution with which the lancet should be used in those inflammations arising from poisoned wounds received in dissection—and also in erysipelatous inflammations, which are nearly allied to them. But we never dreamt that venesection should be withheld where the excitement ran high—where the patient was robust—and especially where the case came under management during the first day or two of the fever. Under such circumstances it would be worse than madness to allow the febrile excitement to ravage uncontrolled on important organs, because a great debility might succeed. That debility must necessarily follow the super-excitement, and probably with such derangement in the vital organs as will render it a much more formidable enemy than the debility resulting from depletion. This is no theoretical view. It is the view which is taken by most of those who are conversant with disease at the bed-side of sickness. We are convinced that no general rule can be even *generally applicable* in the employment or witholding of the lancet in this disease. Not only must each case be managed according to the phenomena which it exhibits, but the same case will require, or not admit venesection, according to the length of time which has elapsed from the commencement of the excitement.

And here we cannot help noticing a passage in the parallel which Dr. Butter draws between the cases of Professor Dease and Dr. Bell, which is, to say the least of it, very erroneous—if not unjustifiable. It is this:—"Pain in the shoulder was felt by Professor Dease on the second day—and by Dr. Bell on the third day. The former (Professor D.) had one hundred leeches applied to the shoulder, and the latter (Dr. B.) was blooded a *second time* from the arm, on the *third day*," p. 76. Now if the reader will turn to the case, he will see that Dr. Bell first complained on the 20th September and was bled on the *same day*. On the 21st, being the *second day* only, he was bled a second time, and not on the *third day* as stated. One day makes an amazing difference in a disease which commenced on the 20th, and terminated fatally on the 24th. Venesection might be perfectly justifiable on the first and second days of a disease, which would be death on the third day. The fact is, that Dr. Bell was bled on the evening of the day of attack, but in so small a stream and quantity that no good was done, and the physicians were obliged to bleed next morning from the extreme urgency of the symptoms. He was bled neither too freely nor too late, as the printed history would lead one to suppose—and as it was, we learnt at the time of his illness and death, and long before Dr. Butter's account was published, that Dr. B. evinced, towards the close of the disease, snorting respiration, fixed staring eyes, and all the usual symptoms of cerebral turgescence. His fever had all the irremediable activity, and all the rapidity of transition from great excitement to prostrate collapse, which characterise the severest fevers of the Torrid Zone. Add to this, that Dr. Bell was the worst of patients—*a medical man!* He was, according to all accounts, distressed and depressed to the last degree, at the moment of attack, in consequence of the cases he had lost—so much so, that he gave himself up to despair, and persisted in saying he should die from the very invasion. Whoever has had the misfortune to have the management of such patients, can know and feel the difficulties and the dangers attendant on such charges! For our parts, we sincerely pity those who have such responsibility thrown on their shoulders, and very cautious should people be in judging of such cases by mere hearsay evidence, or even from histories on paper, drawn up from memory.

We deem it but justice to those concerned, to introduce in this place a detailed statement of one of these cases drawn up on the spot, and by an eye-witness of the phenomena presented. It is the case of Richard Quick, published by Dr. Butter, at page 118 of his work, and occupying just 24 lines. The detail of the case is given by Mr. Tripe, who had the charge of the patient, and published in the Medical and Physical Journal for September 1825.

"*Case.* Richard Quick, (in Dr. B's. work *John*) a smith of his Majesty's dock-yard, aged 38, a man of regular, temperate habits, and cheerful disposition, had been for two years a subject of chronic visceral disease, but had latterly enjoyed a robust state of health. He returned from his duty on the evening of the 21st of September last, much indisposed; and the next morning he took a saline purgative, and absented himself from his labour. His indisposition continued to increase, and on the morning of the 23d I was requested to visit him. His face was much flushed; skin hot; pulse quick and full; tongue moist, and covered with a whitish coat. He complained of excessive pain and throbbing of his head, with weight in the occiput, and of great pain and tenderness in the right hypochondrium. The conjunctivæ were suffused, and all his movements and expressions were indicative of high cerebral irritation. His bowels had been effectively acted upon by the salts which he took the day before.

"He was immediately bled until the force of the circulation was subdued, and an approach to faintness was indicated; when his feelings were generally relieved, and the distress and pain of his head entirely removed. He was directed to take calomel, with tartarised antimony, every six hours, and a solution of sulphate of magnesia.

"24th. No return of pain or throbbing of the head since yesterday; heat of skin decreased; pulse greatly reduced in frequency and force; pain and tenderness of hypochondrium also relieved. Considers himself very much better, in every particular, than on the preceding day. Yet, notwithstanding this general improvement, there still exists some evidences of cerebral irritation: his mind appears to be more than commonly susceptible of impressions, and, in communicating them, he expresses himself with unusual quickness. Medicines have acted well; the excretions are very dark and fetid.

"Infusion of senna, with sulphate of magnesia, to be taken directly; and the pills to be continued, with a mixture containing the liquor ammoniæ acetatis; a blister to be applied to the nape of the neck.

"25th. Has refused to apply the blister, or to take his medicines. Has been in the strongest irritation of mind, with almost every impression delusive; his conduct was occasionally violent, and he exclaimed against me in particular, (for whom he had always, during a long professional acquaintance, appeared to entertain the warmest respect,) and charged me with having intentionally omitted that treatment which had proved so beneficial on the first day of my attendance; adding, that the pain had been thereby removed into both his legs, in which he at this time suffered considerable agony. He vehemently declared that he would submit to no further treatment. To repeated inquiries, he answered that he had no pain of his head or side. The skin and tongue did not exhibit any increase of morbid appearance, but the pulse was advanced in frequency and fulness. No alvine discharges.

"A red patch, about the circumference of a crown-piece, has shown itself upon the outside of the calf of the right leg; and, on examining the limb, I find there has been a recent injury of the great toe, with the loss of the nail; but, on the most careful investigation, I cannot discover any unsoundness, or produce the least uneasiness by pressure. The entire line from the spot upon the calf to the toe is without increased heat, redness, tumefaction, or preternatural sensibility. The following history of the hurt was communicated to me:—A month had elapsed since the great toe was injured by a piece of iron falling upon it, while at work in the dock-yard, for which the surgeons of that establishment attended him; and, at the end of a fortnight, he returned to his employment, cured, and regularly followed his work until the 21st of September, when, it has already been stated, he returned to his home indisposed. In the course of the last day, while at his work, he frequently complained of uneasiness in his toe, and he had removed his stocking to examine it, when he pressed a little matter from one corner. This is the only evidence, however, which we have that he felt the least inconvenience in or near the original hurt, throughout any part of the case.

"In the course of the afternoon (25th) he became tranquil, and his impressions quite correct: he recollected what had passed in the morning, and felt much distressed lest his conduct towards me (which he considered had been disrespectful,) might have offended me. He expressed the most earnest desire that I should retain no resentment; and he entreated that the efforts to relieve him might be renewed, making an exception only to the blister, the application of which he resisted in the most positive manner. He declares himself free from pain in his head and side, but describes the pain in the extremities to be most intense. There is no alteration in the appearance of the spot on the leg, the toe, or any intermediate part between them.—The purgative was ordered to be taken directly, and the pills and saline mixture as last directed; the toe to be enveloped in a bread-and-water cataplasm, and the legs to be bathed in the decoction of poppies.

"26th. Had some sleep, and altogether passed an easier night. Tongue coated as before; pulse undiminished in frequency and force; pain no where but in the legs, which continues equally severe; mind and general feelings nearly in the same condition as last evening. A second red patch has made its appearance upon the outside of the thigh. Medicines have acted well; the excretions continue very dark and fetid.—The medicines, cataplasm, and fomentations, were continued.

"27th. Perfectly incoherent; exhibits great irascibility, varied with levity, and occasional violence of expression and gesture. Does not complain of pain in any part, excepting in the right leg, which is swollen in the calf, with great increase of redness and heat: the least pressure or motion of the limb occasions great distress. Other patches, similar in appearance to those upon the leg and thigh, have made their appearance upon the opposite side of the body, on the right arm, and various parts of the trunk. The tongue and pulse still maintain a similar character, and the excretions continue very vitiated. His saline medicine to be continued, with camphor mixture, and his pills, with the addition of opium. The purgative to be repeated; and the right leg was directed to be enveloped in a yeast cataplasm.

"28th, nine A.M. Has passed a most distressing night, without any quiet or lucid interval. The pulse is very quick, slightly irregular, and less forcible; occasional subsultus; tongue not materially changed; swelling, redness, and tenderness of the limb extending down to the foot, where there are some appearances of vesication, and upwards to the knee. The spots on the thigh and on the arm are increased. Dr. Magrath visited him this morning, but he refused from this period every kind of medicine.

"Nine P.M. Since our last visit he has become furious: whilst I was with him, he forced himself out of bed, where he continued more than half an hour; and, in the attempt to return him to his bed, he resisted, and made a powerful effort to pull us under the bedstead, in which he persisted; and he continued, with loud shrieking, to struggle with the united efforts of three or four strong men for a considerable time, before he sunk exhausted. The swelling of the limb is much increased; the vesications are larger and more numerous; many of them are broken, and discharge a bloody serum: the fore part of the foot is livid.

"29th. Has continued violently delirious all night; is now completely comatose, with dilated pupils, tremulous pulse, subsultus tendinum, laborious and stertorous breathing. These symptoms continued until midnight, when he expired.

"*Examination of the Body, twelve hours after Death.* There were present my apprentices, and Mr. J. G. Sparke, a surgeon of Devonport, who kindly assisted me.

"Skin deeply tinctured with bile over the upper parts of the trunk and face. Muscles of the face distorted, and expressive of severe bodily sufferings. Effluvia highly offensive and pungent. Extensive gangrene covering the anterior part of the right foot and leg, which was much swollen.

"More than ordinary exertion required in turning back the scalp; the connecting cellular membrane between it and the pericranium having become so dense as to nearly bring them into union. Much blood effused, from numerous and larger vessels than usual being divided in performing this part of the operation. On the calvaria being forced off, the ruptured vessels in the course of the superior longitudinal sinus bled copiously. The vessels of the dura mater, and the superior longitudinal sinus, were completely gorged. On the removal of the dura mater, a large quantity of serum escaped. The cerebrum appeared as if enveloped with a stratum of coagulable lymph, the third of an inch in thickness, which, being cut into, proved to be a collection of serum, distending the tunica arachnoidea. The superficial veins of the cerebrum were loaded. Medullary substance of the cerebrum, from the surface to the corpus callosum, very dense and solid. The divided vessels, on making the different sections of it, presented extravasated spots of blood, much more expansive and numerous than ordinary. The centrum ovale greatly distended, and, on being cut into, gave exit to a large quantity of serum from the lateral ventricles, which, with the other cavities, were greatly enlarged by the contained fluid. Plexus choroides, and investing membranes throughout the ventricles, highly injected.

"No appearance beneath the tentorium worthy of particular observation, excepting that the cerebellum partook of the unusual hardness which characterised the cerebrum, and that the lateral sinuses and veins were gorged with blood.

"Some old pleuritic adhesions were rather extensive on the right side of the thorax.

"In the abdomen, the liver, larger than ordinary, had formed adhesions to the right side; its structure more dense than natural, particularly along the anterior margin of the right lobe; its surface pallid, but its interior filled with blood. Gall-bladder distended with bile. The villous coat of the stomach was inflamed throughout, and was covered with spots of ecchymosis. The left kidney twice the natural size; its shape and structural arrangement of regular appearance. The right kidney so wasted and obscured by disease, that it could no where be felt, but was, after some search, traced (by the shrunk ureter) lying high up under the liver, and was with much difficulty removed, from the intimate connexion which it had formed, by its thickened cellular coat, to all the surrounding parts. Its organisation was destroyed; it had a schirrous feel, and was loaded with calculous matter.

"The intestines exhibited no morbid appearance, either upon their inner or outer surfaces, at the different points that were examined. Spleen, pancreas, and urinary bladder healthy. Peritoneum generally free from morbid character.

"The parts beneath the cicatrix of the injured toe, from the surface to the bone, were perfectly free from any evidence of disease; nor did it exhibit any proofs that the bone had suffered an injury from the original accident. The cellular membrane investing the tendon of the extensor proprius, about the extremity of the second bone of the great toe, was injected with a yellow and bloody serum. This appearance did not extend in the course of the tendon, but towards the surface, and was soon involved in the general disease of the limb. The fore part of the foot, and lower part of the leg, extensively sphacelated. The cellular substance about the middle and outside of the leg, and extending down to the fascia, much swollen, and filled with bloody serum, and intersected with small cavities containing pus. The texture of the muscles between this and the bones completely destroyed, and converted into a dark-coloured mass, not unlike putrid placenta. An incision made into the cellular substance beneath the patch on the arm of the opposite side, gave exit to a quantity of greyish, unhealthy looking pus. The other spots were not opened, their appearance being precisely similar." 180.

We are tempted to give the other case published by Mr. Tripe, because the subject is of extreme importance, and we are anxious that the public should have the fullest evidence on which to ground their decision as to the proper treatment.

*Case.* "On the 2d of October, ten P.M. I was requested by Mr. Dryden, the assistant-surgeon of the dock-yard, who was now in charge of that establishment, in consequence of the death of the surgeon, to visit with him John Walkie, shipwright, a married man, aged thirty-eight, spare habit, sallow complexion, regular in his mode of living, and enjoying good health. Previously to my seeing the patient, Mr. Dryden informed me that Walkie had received a slight hurt upon his right leg ten days before, which had thrown off a superficial eschar, leaving a well-conditioned ulcer, that had been dressed in the usual manner with adhesive straps, on the 30th September. In the evening of the 1st of October, he complained of uneasiness in his leg, and was soon seized with rigors, which were succeeded by heat and pain of his head. The dressings on his leg were then exchanged for a warm cataplasm, and he took aperient medicine. On the morning of the 2nd (being the day on which Mr. Dryden called upon me) the symptoms continued, and there was prescribed for him calomel, and a repetition of the purgative. These operated well; but at three, P.M. the pain of head and fever had increased, and he lost twenty-five ounces of blood, which was followed by a mitigation of his complaints. In the course of the evening, the activity of symptoms having recovered itself, twelve leeches were applied to the temples, and the orifices were bleeding at ten o'clock, when we visited him. He was complaining of great distress of his head, with weight of the occiput, and lancinating pains through to the forehead. He had a flushed anxious countenance, and he expressed himself with fearful apprehension; he had violent throbbing of all the vessels of the head, and those of the conjunctiva were highly injected with blood. He had a sense of weight at the præcordia, which affected his respiration; and he experienced much uneasiness by pressure all around the body, from the right to the left hypochondrium; he had uneasiness in discharging his urine, and he complained of pain above the pubis. Pulse 120 and full, and the heat of the body increased; tongue was slightly coated, whitish, with an approach to brown in the centre. The ulcer upon his leg was about the size of the little finger nail; it had a clean granulated surface, but rather pallid; there was no heat or redness around it, or in any part above it; he shrunk, however, from pressure made along the course of the absorbents, from the wound up to the inguinal glands, which were enlarged, and appeared to be the most sensitive point. Blood, which was drawn in the afternoon, exhibited slight spots of buff.

"Venæsectio repetatur ad ℥xxviii., which very sensibly reduced the forcible action of the heart and arteries, and almost totally removed the suffusion of the eyes, with decided amelioration in all the feelings of the patient. He was directed to take ten grains of calomel, and to repeat three grains every second hour; to have a purging enema injected at five in the morning. The head to be shaved, and kept cool by the application of cloths wetted in a mixture of liquor ammoniæ acetatis and spiritus vini. The body, whenever heated, to be sponged with vinegar.

"3rd, eight A. M. — Countenance depressed; yellowness of the skin; dullness of the eye; flushing of the right cheek; tongue inclined to dryness towards the point, and more approaching to brown; pulse very frequent and small. Head quite relieved, as well as the pain around the body at the scrobiculus cordis, and the difficulty of breathing. Complains of increased pain in the hypogastrium, which is excessively tender from the right to the left inguen, but most so in the right. There is a sense of tightness across this part, and the distress is increased upon every deep inspiration. No swelling or redness is observable in the course of the absorbents, from the ulcer to the inguinal glands, but the latter are rather more enlarged. Blood, drawn last night in a wine glass, is covered with a thick stratum of coagulable lymph, and the crassamentum of that which was drawn in a basin is dense and heavy. A vein was again opened, and about thirty ounces drawn. During the flow the pulse gradually rose, until it became full and expansive; and it was not until this quantity was extracted, that it was deprived of a force which we considered unsafe. After this he had a very large discharge of flatus, with some dark-coloured feculent matter, being the first evacuation since the enema was injected. He said he was greatly relieved, and the improvement in his countenance corresponded with his declaration.

"Four grains of opium to be taken directly; and a pill every two hours afterwards, composed of calomel, tartarized antimony, and opium. The enema to be repeated.

"Eleven, A.M.—Mr. Dryden visited him, and finding his pulse full and hard, with considerable return of pain, ordered twenty-four leeches to be applied to the hypogastrium.

"One P.M.—Leeches have bled well. The patient says he has derived very great relief from them, and expresses himself, in consequence, with confidence of his recovery. He can bear pressure upon the groins with comparative ease. Pulse ninety, equable and soft. Blood drawn this morning cupped, and extensively buffed; crassamentum firm and tough, requiring very considerable force to break down its texture.

"Eight P.M.—Has had some refreshing sleep, and still doses; has had two dark-coloured stools: says he is much easier and better in all his feelings, but has some lingering pains occasionally in the lower part of the abdomen. Countenance is pallid, but more tranquil; body covered with a warm perspiration; pulse 120, soft and regular; dryness and brown colouring of the tongue increased, (probably the effect of the opium.)—Directed a blister to be applied to the hypogastrium; the pills to be continued, and the injections repeated.

"4th, eight A.M.—Mr. Dryden reported that all his symptoms were ameliorated, and his pulse 108. I was prevented from seeing him at his hour by indisposition.

"Eleven A.M.—Visited him. He had enjoyed short sleeps throughout the night; his countenance was improved, and his spirits good. Tongue moist, and cleaned around the edges; pulse 120, regular and firm; has an occasional eructation, resembling hiccough, and still complains of pain above the pubis, with tightness in taking a full inspiration. Injections operated well, and the blister fully vesicated. Directed him to have twenty-four leeches applied to the blistered surface, and afterwards to be put into a warm bath. Pills and injection as before.

"Nine P.M.—Leeches have sucked well, and he has been in the bath twenty minutes without the least faintness. A violent hiccough distressed him for an hour and a half before he went into the bath, but it was entirely removed by it; and, soon after being replaced in his bed, a profuse warm perspiration broke out, which still continues. Has now no pain in any part of the abdomen; can bear pressure or full inspiration, without any uneasiness. Tongue moist, and nearly cleaned; pulse 120, expansive and regular; countenance good; mind inspired with confidence.—Directed the pills to be continued; the bleeding from the leech-bites to be arrested, and eighty drops of tincture of opium, in a draught to be taken, if the singultus should return.

"5th, seven A.M.—Was prevented from repeating my visit at this hour by indisposition. Mr. Dryden reported him to be sinking, with cold extremities, sunken pulse, anxious breathing, stools mixed with blood—senses entire.—Directed him to have warm wine, camphor mixture, with carbonate of ammonia: and the heat of the body to be sustained by bottles of warm water, &c.

"Eleven A.M.—I visited him, and found him as above, with occasional discharges of dark-coloured blood from the anus. The leech-bites were still bleeding.

"Twelve A.M.—Again visited him, and was informed that he had awoke out of his stupor in a state of delirium, and insisted upon getting out of bed; he exhibited so much appearance of determination, that the female attendants in the room deemed it right to call for assistance to restrain him. He had now, however, become perfectly calm, and restored to his senses. He continued undisturbed until his dissolution, at a late hour in the evening, having through the day occasional discharges of blood.

"*Dissection, about twenty hours after death.*—Mr. Dryden examined the body, and reported to me the following appearances;—slight evidences of inflammation upon the surface of the small intestines; also a patch of redness around the cardiac orifice of the stomach. Redness about the peritoneal lining of the pelvis, and effusion of some puriform fluid into that cavity. The limb exhibited no appearance of disease any where in the course of the absorbents, excepting a very small sinus about an inch above the wound, but which contained no fluid. I was prevented from being present at the dissection, in consequence of my indisposition having so increased as to render me at that time incapable of any exertion. I deeply regret that it was not thought necessary to examine the brain; and the same omission in the other cases is equally to be lamented. This most important viscus formed a point, in all the cases, on which the disease made its earliest attack, and against which it continued to direct a considerable part of its force. Quick's was the only case in which the brain was examined, and the appearances of diseased action exhibited in that organ afford ample reason for this regret in the others." 183.

We have now laid before the reader ample specimens of this very formidable disease, to enable him to form some judgment of its nature. Before coming to the subject of treatment, we shall say a few words respecting its etiology. The teak wood used in the Dock-yard was, at one time, suspected to be the cause, but the idea is perfectly ridiculous; then the dressings employed at the surgery. The air was, with more reason, suspected of producing a constitutional tendency to erysipelatous inflammation—and the truth is, that such cause does exist in the air, or in the earth: but how developed, and of what elements composed, we are perfectly ignorant. It is true, as Dr. Butter says, "the air of Plymouth is mild, salubrious, and beneficial to invalids;" but then we know that the healthiest spots in the world become occasionally unhealthy, and without any assignable cause. We cannot, therefore, conceive any *general* cause of this morbid susceptibility, except what is produced by the elements around us. There are, doubtless, many *local* or *individual* causes, as inebriety, mental depression, and the like, but these never could account for an epidemic influence. It is hardly necessary to observe, that this tendency to erysipelatous inflammation and atonic fever prevailed in several parts of England and Scotland nearly about the same time that the disease was producing so much mischief at Devonport.

*Treatment.* On this subject, however important, we cannot dwell long. Our sentiments on this head are already known—having been expressed in the reviews of Dr. Duncan's important paper on diffuse inflammation of the cellular membrane, and of several papers in the Dublin Transactions, and in various periodical journals. Every practitioner who has had much clinical experience, must be convinced that the treatment of each individual case must be regulated by the constitution, the attendant circumstances, and the actual symptoms present. Our own observations would lead us to be more cautious of the lancet than one of the authors at the head of this article—Mr. Tripe; but much less fearful of it than the other—Dr. Butter. The didactic precepts laid down by the latter author are, in our opinion, more judicious than the incidental remarks on the individual cases. On this account, we shall give a slight sketch of Dr. B's chapter on the methodus medendi.

He divides the remedial agents properly into local and general. Of the local, we are disposed to agree with him in condemning cold and repellant lotions as topical applications to an erratic inflammation like the present. Fomentations and poultices are far more likely to be of service in such cases. To deep incisions we are friendly, especially when there is reason to believe that the cellular membrane is inflamed, and likely to suppurate. We have often practised them, and seen their good effects.

In respect to general bleeding, although the tenor of Dr. Butter's remarks, en passant, on the cases themselves, would deter any young practitioner from this measure, in toto, yet he observes, at page 244, "I do not mean to deny that venesection may *not* (superfluous) be employed with manifest advantage occasionally, especially where there is (are) a full bounding pulse, a hot skin, and other strong indications of plethora; but even here I would bleed with caution." The symptoms abovementioned were the ipsissima symptomata presented by the late Dr. Bell, when his medical attendants advised the detraction of blood—nor was the quantity large, nor the stage of the disease advanced, at the time.

"In the disease under consideration, there were the symptoms of pyrexiæ, of neuroses, and of cachexiæ, of all three classes combined; and, therefore, highly as I revere the departed genius of Dr. Gregory, I think that his views were moulded to the Cullenian system, and fashioned by scholastic restraint.

"At the commencement of irritative fever, when the head is greatly affected, especially in a plethoric person, I might be disposed to advise the loss of some blood, either from a vein or topically, with the view of relieving the system, as Mr. Hunter supposed, '*mechanically;*' but this principle of acting differs from that of a person who would say—'Sir, this is an inflammatory disease, and bleeding is the remedy.'" 246.

In this, and indeed in many other observations, we are disposed to agree with our author. We should only employ venesection, as we said before, with the view of controlling that severity of excitement which threatened the function or structure of important organs, always bearing in mind, that a heavy subsequent demand is to be made on the constitution, and that its powers are to be economized, and not wasted, previously to this last struggle between nature and disease. This is a rule which we have always inculcated, when speaking of the disease in question. In consonance with this rule, we should not be inclined to recommend beef-steaks and porter with Mr. Shaw, nor bleed away at so furious a rate as Mr. Tripe does. Upon the whole, we would rather follow the precepts of Dr. Butter than those of either of the opposite parties.

In respect to local bleeding, followed by fomentations, there can be little doubt of the propriety of this measure. Leeches may also be applied to relieve the head or other organ threatened, when the safety of general bleeding might be problematical.

Dr. Butter's remarks on the various other medicines which are used in irritative fever and erysipelatous inflammation, as bark, mercury, antimony, ammonia, brandy, &c. need no notice in this place, as they do not appear to rest on any extensive personal observation or experience in the disease under consideration. In closing this article we are forced to observe, that the author frequently travels far out of the record, and diverges to subjects which have a very remote connexion indeed with the main one on which the volume is founded. It would have been more advantageous to himself, as well as the public, if he had kept to his text, and avoided so many unnecessary digressions, by which the work is swelled to an extent that will greatly abridge its circulation, and thus frustrate the object of the writer. In other respects, the volume is indicative of intelligence, erudition, good sense, and considerable critical acumen. We part from the author with sentiments of respect and esteem.

